# Broken silence: 22,841 predicted deleterious synonymous variants identified in the human exome through computational analysis

**DOI:** 10.1590/1678-4685-GMB-2023-0125

**Published:** 2024-01-22

**Authors:** Ana Carolina Mello, Delva Leao, Luis Dias, Felipe Colombelli, Mariana Recamonde-Mendoza, Andreia Carina Turchetto-Zolet, Ursula Matte

**Affiliations:** 1 Hospital de Clínicas de Porto Alegre, Núcleo de Bioinformática, Porto Alegre, RS, Brazil; 2 Hospital de Clínicas de Porto Alegre, Centro de Pesquisa Experimental, Laboratório de Células, Tecidos e Genes, Porto Alegre, RS, Brazil; 3 Universidade Federal do Rio Grande do Sul, Programa de Pós-Graduação em Genética e Biologia Molecular, Porto Alegre, RS, Brazil; 4 Universidade Federal do Rio Grande do Sul, Programa de Pós-Graduação em Ciências Biológicas: Bioquímica, Porto Alegre, RS, Brazil; 5 Universidade Federal do Rio Grande do Sul, Instituto de Informática, Porto Alegre, RS, Brazil; 6 Universidade Federal do Rio Grande do Sul, Departamento de Genética, Porto Alegre, RS, Brazil

**Keywords:** Synonymous variants, deleterious, human exome, bioinformatics

## Abstract

Synonymous single nucleotide variants (sSNVs) do not alter the primary structure of a protein, thus it was previously accepted that they were neutral. Recently, several studies demonstrated their significance to a range of diseases. Still, variant prioritization strategies lack focus on sSNVs. Here, we identified 22,841 deleterious synonymous variants in 125,748 human exomes using two *in silico* predictors (SilVA and CADD). While 98.2% of synonymous variants are classified as neutral, 1.8% are predicted to be deleterious, yielding an average of 9.82 neutral and 0.18 deleterious sSNVs per exome. Further investigation of prediction features via Heterogeneous Ensemble Feature Selection revealed that impact on amino acid sequence and conservation carry the most weight for a deleterious prediction. Thirty nine detrimental sSNVs are not rare and are located on disease associated genes. Ten distinct putatively non-deleterious sSNVs are likely to be under positive selection in the North-Western European and East Asian populations. Taken together our analysis gives voice to the so-called silent mutations as we propose a robust framework for evaluating the deleteriousness of sSNVs in variant prioritization studies.

## Introduction

Point mutations in protein coding sequences may lead to remarkable functional changes and their severity can be classified by evaluating the extent of amino acid alterations to protein function (Cooper *et al*., 2010). Synonymous single nucleotide variants (sSNVs) do not alter the sequence of amino acids due to codon degeneracy, seemingly causing no change to protein function. Because of this, sSNVs are often discarded in variant prioritization pipelines (Buske *et al*., 2015).

The idea that sSNVs are innocuous has been recently challenged when several studies associated these variants to different diseases (Gartner *et al*., 2013; Bonin *et al*., 2016; Diederichs *et al*., 2016; Palagano *et al*., 2017). sSNVs in GWAS studies share similar likelihood and effect size to disease association as non-synonymous SNVs (Chen *et al*., 2010). The mechanisms by which sSNVs can cause deleterious consequences comprise a series of processes related to modulation of gene expression, such as aberrant splicing (Cartegni *et al*., 2002), modified mRNA stability (Nackley *et al*., 2006) and changes in the pace of synthesis and cotranslational folding of proteins due to codon usage bias (Hunt *et al*., 2014).

Studies on yeast have demonstrated that the majority of synonymous variants are strongly nonneutral and can have significant effects on gene expression levels (Shen *et al*., 2022), suggesting that synonymous variants may play a more important role in shaping an organism’s phenotype than previously thought. Despite the demonstrated importance of sSNVs, efforts to experimentally elucidate the functional consequences of sSNVs are scarce, especially when compared to initiatives validating non-synonymous variants (Buske *et al*., 2015). For such, while methods for predicting the consequence of sSNVs to protein function have been developed (Buske *et al*., 2015), we lack a robust framework for evaluating sSNVs deleteriousness to the benefit of variant prioritization studies.

Here, we sought out to develop a framework to assist in the deleteriousness prediction of sSNVs identified by whole exome sequencing (WES) data. We obtained candidate deleterious (detrimental) sSNVs by combining the prediction results of Silent Variant Analysis (SilVA) and Annotation Dependent Depletion (CADD). Next, we evaluated the weight of features to deleteriousness classification via Heterogeneous Ensemble Feature Selection, and comprehensively analyzed the frequency of variants and gene ontology. Finally, we evaluated if benign variants could be subject to positive selection using the Population Branch Statistics (PBS), an F_st_ based method.

## Material and Methods

### Dataset

All the data used here are publicly available on The Genome Aggregation Database (gnomAD v2.1.1) (Karczewski *et al*., 2020). We downloaded the variant call format (.vcf) files for all 24 human chromosomes, separately, containing data from 125,748 exomes, all mapped to the GRCh37/hg19 reference sequence. The Y chromosome was cut out from the analyses because one of the prediction softwares (SilVA) doesn’t have support for this chromosome.

### Synonymous variants identification and effect prediction

Each .vcf file was used as input for SilVA (v1.1.1) (Buske *et al*., 2015), which identifies only synonymous variants and predicts their effects. SilVA bases its predictions on a number of features, including conservation, codon usage, splice sites, splicing enhancers and suppressors, and mRNA folding free energy. We used all variants classified as synonymous by SilVA as input to CADD (v1.4) (Rentzsch *et al*., 2019), which is a variant effect predictor not specific to sSNVs. CADD integrates multiple annotations into one metric by contrasting variants that survived natural selection with simulated mutations. Next, we selected only the variants classified as synonymous both by SilVA and CADD.

The next filter step involved the effect predicted for each variant. We separated our dataset into two groups: sSNVs predicted as deleterious and sSNVs predicted as benign. The CADD PHRED-like scaled score ranks a variant relative to all possible substitutions of the human genome (8.6x109) (Rentzsch *et al*., 2019). A PHRED-like score greater or equal to 20 indicates the 1% most deleterious, while a score greater or equal to 30 indicates the 0.1% most deleterious. For this study, variants with a PHRED-like score ≥ 15 were considered as detrimental and variants with a PHRED-like score < 10 were considered as benign, as recommended by the authors. On the other hand, SilVA classifies the variants as benign (score ≤ 0.270), potentially pathogenic (0.270 > score ≤ 0.485) and likely pathogenic (score > 0.485) based on the predicted score that ranges from 0 to 1, where close to 1 is more likely to be deleterious. Variants featuring both pathogenic SilVA classes (potentially and likely) were considered as deleterious for this work. Variants which had divergent effect prediction between SilVA and CADD were filtered out and the remaining sSNVs composed our final dataset.

### Ensemble feature selection

To find out which features contributed the most to the prediction of detrimental variants, we performed a Heterogeneous Ensemble Feature Selection (EFS) on 53 features from the CADD annotations (Table S1). The remaining 46 features were filtered out for either presenting more than 5% of missing values or not making sense to this specific analysis (Table S2). We combined both Python3 and R programming languages to create an ensemble with four filter methods provided by FSelector R package (Romanski *et al*., 2021): Chi-square (Bommert *et al*., 2020), OneR (Holte, 1993), Gain Ratio (Quinlan, 1986), and Symmetrical Uncertainty (Bommert *et al*., 2020).

All the feature selection methods provide a features ranking, from the most to the least relevant feature to discriminate among classes (i.e., deleterious or benign) based on scores computed according to their particularities. Our ensemble setup combines the four different feature relevance opinions using the Borda Count method, a popular voting rule that combines preferences of multiple voters. Due to the severe imbalance between the number of deleterious and benign variants in our dataset, we performed an undersampling on the benign data, which is the majority class. We randomly sampled the benign class 100 times for the same amount of variants in the deleterious class and performed the Ensemble Feature Selection with all samples. The final ranking was a combination of all 100 rankings also using the Borda Count method.

### Gene enrichment

To investigate the functions of the genes on which the detrimental sSNVs are located and the biological pathways implicated, we developed the R package called autoGO as an integrator of gene enrichment for Gene Ontology (GO) and Kyoto Encyclopedia of Genes and Genomes (KEGG) pathways. The GO search depends on the package clusterProfiler (v3.18.1) (Yu *et al*., 2012) and the enrichment of KEGG pathways depends on the package KEGGprofile (v1.32.0) (Zhao *et al*, 2020). ClusterProfiler allows the user to perform the KEGG pathways enrichment as well, however, it uses a deprecated version of the KEGG database, while KEGGprofile connects with the up-to-date data available online. Both packages perform hypergeometric tests to assess the significance of the enrichment followed by false discovery rate (FDR) correction for multiple comparisons.

As the advantages over the currently available packages, autoGO was designed to deal with files containing numerous genes, working as a standalone application. AutoGO performs the analysis from a simple table containing the gene identifiers with or without expression data, generating standardized plots and tables for each input file, regardless of the source of the enriched terms. Consequently, AutoGO can optimize the efforts on the analysis of genomics data. Sources may be found at https://github.com/ldiass/MPSbase/tree/master/autoGO.

### Identification of outliers

In order to evaluate the possibility of positive selection acting upon the benign variants dataset, we used the F_st_ based Population Branch Statistics (PBS) to detect outliers at first. Introduced by Wright (1951) as part of the F-statistics, the F_st_ is a descriptive measure of the differentiation prompted by important evolutionary processes such as migration, mutation and drift, between two populations (Holsinger and Weir, 2009). Since F_st_ is directly related to the variance in allele frequency, small F_st_ values indicate similarity of the allele frequencies within each population.

PBS was first described by Yi *et al*. (2010) in a study of heritable adaptations to extreme altitude in the Tibetan Plateau population. It is based on the premise that a gene presenting large differences in allele frequencies between two populations configures a potential target for natural selection. However, simply ranking F_st_ values wouldn’t tell which population was affected by selection. PBS introduces a third more distant population for a pairwise F_st_ comparison, yielding in the PBS value. Variants which present extreme PBS values - the outliers - configure strong candidates for positive selection in the first population. Here, we developed an R script to calculate PBS values as described by Yi *et al*. (2010).

Two groups of three populations were separately analyzed: East/South Asian (EAS-SAS), and North-Western/South European (NWE-SEU), with the third and more distant population being the African/African American for both groups. Population datasets were obtained from GnomAD using the database’s classification. These populations were chosen for the similarities between the natives from the same continent given by the minimal F_st_ values between them (EAS-SAS = 0.00108 and NWE-SEU = 0.00029), and for the blatant differences from the African/African American natives. The African/African American population is represented by 8.128 individuals, South Asian by 15.308, East Asian by 9.197, whereas South and North-Western European are represented by 56.885 individuals together.

As a support analysis, we separately ran the same groups of populations on Bayescan (v2.1) using default parameters. Just like PBS, Bayescan is an F_st_ based method used to identify variants under natural selection. The difference is that it uses a Bayesian model for estimation of locus-population specific F_st_ coefficients.

## Results

### The majority of human synonymous variants are predicted to be neutral

Our raw dataset consisted of 16,754,528 single nucleotide variants found in 125,748 exomes (Karczewski *et al*., 2020). After combining prediction results from SilVA and CADD, we obtained 1,266,032 sSNVs, corresponding to 7.56% of our raw dataset. Out of this set of sSNVs, 98.2% (1,243,191 variants) were considered benign (File S1), while 1.8% (22,841 variants) were predicted to be deleterious by both predictors (File S2), yielding an average of 0.18 deleterious sSNVs per exome (Figure S1). In contrast to studies on yeasts (Shen *et al*., 2022), the majority of synonymous variants found in humans are predicted to be neutral.

**Figure 1 -:**
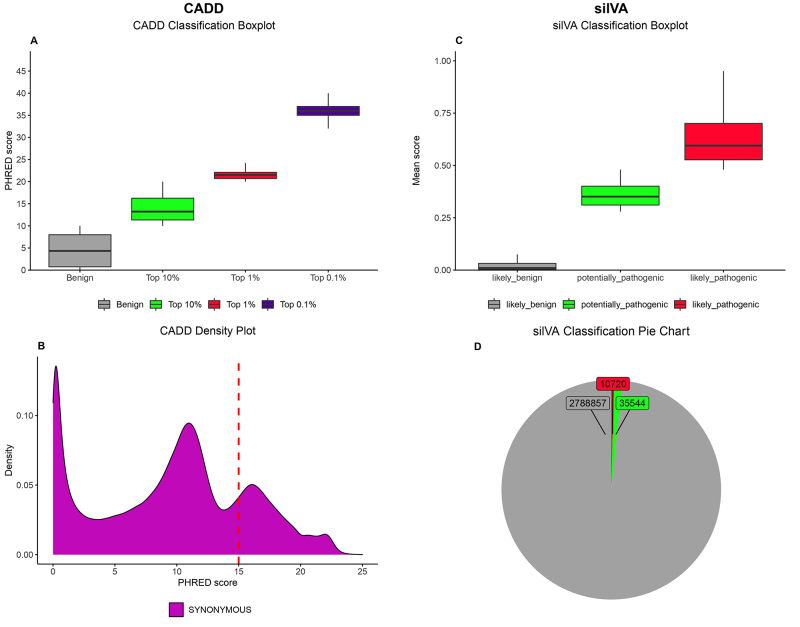
Comparison between SilVA and CADD predictions. **A.** CADD’s prediction boxplot. In grey, the benign group, in green the top 10% most deleterious variants, in red the top 1%, and in blue the top 0,1%. The Y axis indicates the PHRED-like scaled score; **B.** Density plot of CADD’s prediction. The dashed red line indicates the cutoff for deleterious variants **C.** SilVA prediction boxplot. In gray, the likely benign group, in green, the potentially pathogenic group, in red, the likely pathogenic group. Y axis indicates the mean SilVA score; **D.** Pie chart of SilVA’s prediction. Same color code as Figure 1C .

CADD ranked 589,360 variants as the top 15% deleteriousness, which corresponds to its cutoff. Only 186 variants were ranked as the top 0.1% most deleterious (Figure 1A and 1B) with a PHRED-like score > 30. Out of 46,264 variants predicted as deleterious by SilVA, 10,720 sSNVs fell in the likely pathogenic category, with scores higher than 0.48, and only 77 obtained a score higher than 0.9 (Figure 1C and 1D). None of the 77 top-scoring variants from SilVA’s prediction are ranked among the 0.1% CADD variants.

The sSNV 9-139685876-G-A is the only featuring CADD’s top 5 scoring variants that was predicted as likely pathogenic by SilVA, with a 0.586 score. The remaining four variants featuring CADDs top 5 were considered potentially pathogenic by SilVA, with a score ≤ 0.350: 19-7747163-G-T, 20-35807771-G-A, 3-73047268-G-A, 9-35809402-G-C. Four out of five SilVA’s top 5 variants are included in the 1% predicted as most deleterious by CADD (Table 1). 

**Table 1 -:** Top-scoring pathogenic variants from each predictor and their strongest gene-disease association according to MalaCards.

Predictor	Variant	CADD*	SilVA*	Gene	Disease (MalaCards ID)
CADD	3-73047268-G-A	44.00	0.299	*PPP4R2*	multiple cancers, SPN046^1^
	19-7747163-G-T	37.00	0.322	*TRAPPC5*	SPN405^2^, BNC002^3^, OST110^4^, RTN041^5^, multiple Cancers
	9-35809402-G-C	33.00	0.350	*SPAG8* ^*§*^	ACR128^6^, EPP011^7^, SHR084^8^
	9-139685876-G-A	32.00	0.586	*TMEM141*	INT004^9^, multiple cancers
	20-35807771-G-A	24.80	0.325	*RPN2*	CNG411^10^, ANM080^11^, multiple Cancers
SilVA	12-105537021-G-A	22.50	0.951	*WASHC4* ^*§*^	INT474^12^, ATS204^13^
	6-152631823-C-T	18.37	0.951	*SYNE1* ^*§*^	SPN207^14^, EMR014^15^, ART165^16^, SPS008^17^, EMR018^18^, JVN050^19^
	5-137488171-C-T	20.30	0.943	*BRD8*	multiple cancers
	8-96047804-G-A	22.30	0.936	*NDUFAF6* ^*§*^	MTC164^20^, FNC066^21^, LGH007^22^, PRM384^23^
	6-31922996-C-T	23.60	0.933	*NELF-E*	ATM095^24^

1 Spinal muscular atrophy. ^2^X-Linked Spondyloepiphyseal Dysplasia Tarda. ^3^Binocular Vision Disease. ^4^Osteogenesis Imperfecta Type Xv. ^5^Retinitis Pigmentosa 11. ^6^Acromesomelic Dysplasia 1. ^7^Epiphyseal Chondrodysplasia, Miura Type. ^8^Short Stature With Nonspecific Skeletal Abnormalities. ^9^Intraneural Perineurioma. ^10^Congenital Disorder of Glycosylation, Type in. ^11^Anemia, Congenital Dyserythropoietic, Type Iiia. ^12^Autosomal Recessive Intellectual Developmental Disorder 43. ^13^Autosomal Recessive Non-Syndromic Intellectual Disability. ^14^Spinocerebellar Ataxia 8. ^15^Autosomal Dominant Emery-Dreifuss Muscular Dystrophy 4. ^16^Myogenic Type Arthrogryposis Multiplex Congenita 3. ^17^Spastic Ataxia. ^18^Autosomal Dominant Emery-Dreifuss Muscular Dystrophy 2. ^19^Juvenile Amyotrophic Lateral Sclerosis. ^20^Mitochondrial Complex I Deficiency, Nuclear Type 17. ^21^Fanconi Renotubular Syndrome 5. ^22^Leigh syndrome. ^23^Primary Fanconi Renotubular Syndrome. ^24^Autoimmune Disease.

§ Gene is likely to be associated with causing the disease(s), since their gene-disease associations are supported by manually curated and trustworthy sources.

* SilVA classifies the variants as benign (score ≤ 0.270), potentially pathogenic (0.270 > score ≤ 0.485) and likely pathogenic (score > 0.485), whereas CADD uses a PHRED-like score system, separating the deleterious category into the 1% most deleterious (PHRED score ≥ 20) and the 0.1% most deleterious (PHRED score ≥ 30). For this study, variants with a PHRED-like score < 10 were considered as benign.

### “Consequence” is the most relevant feature to the effect prediction

Using the comprehensive annotation provided by CADD for each variant (Tables S1 and S2), we performed a Heterogeneous Ensemble Feature Selection in order to rank these annotations according to their relevance to the effect prediction. The top ten most relevant features are related to the type of variant, conservation scores, chromatin state, and the reference amino acid (Table S1). The most relevant feature is ”consequence”. Since we are assessing only synonymous mutations, our final dataset presented only two types of consequences: ”synonymous” and ”splice site” (synonymous variants occurring in splice sites). From the deleterious dataset, 75.72% of the variants occur in splice sites, whereas only 1.12% of benign variants occur in splice sites. Interestingly, 25% of deleterious variants occur outside splicing sites. The remaining features in the top eight are conservation scores given by different softwares. The ninth most relevant feature is the proportion of heterochromatin state in 127 cell types.

### More than 90% of the sSNVs are super rare 

Next, we categorized detrimental sSNVs according to their allele frequency (AF) into not rare, rare and super rare variants. In our data, more than 90% of the variants from both classes, benign and deleterious, present an AF ≤ 0.1% and are considered here as super rare (Figure S2). The proportion of super rare detrimental variants is statistically higher than super rare benign variants, whereas the proportion of rare (0.1% ≥ AF ≤ 1%) and not rare (AF > 1%) deleterious variants is statistically lower than rare and not rare benign variants (p < 2.2x10−16).

**Figure 2 -:**
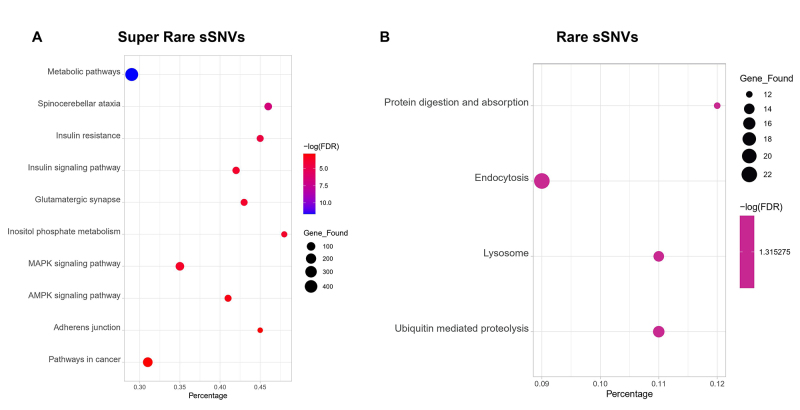
KEGG pathways analysis**. A.** Top-10 KEGG pathways enriched in the super rare variants genes; B. KEGG pathways enriched in the rare variants genes.

Out of the 22,841, 21,926 were considered super rare deleterious variants, located on 9635 different genes. KEGG pathway (Figure 2A; File S3) and Gene Ontology (Figure S3; File S4) enrichment analyses revealed that metabolic pathways are enriched in 438 genes (FDR = 2.12x10−14), corresponding to less than 35% of the total gene set where this pathway is represented. Nevertheless, the spinocerebellar ataxia pathway was found to be enriched in 66 genes (FDR = 2.76x10−9), corresponding to almost 50% of the gene set associated with this pathway. 

The 876 rare deleterious variants are located on 827 different genes, yet only 63 were found to be implicated in four different KEGG pathways (Figure 2B ; File S5) according to enrichment analyses (Figure S4; File S6): Ubiquitin mediated proteolysis (FDR = 0.00039, 15 genes), Lysosome (FDR = 0.00046, 14 genes), Endocytosis (FDR = 0.00056, 22 genes) and Protein digestion and absorption (FDR = 0.00055, 12 genes). The latter is the only pathway not found to be enriched in the super rare deleterious sSNVs genes.

For the 39 not rare deleterious variants, no KEGG pathway was found to be enriched in the 39 different genes on which they are located. Remarkably, all genes have been associated with one or more diseases, with the exception of *GKAP1*, on which the sSNV 9-86354657-A-C is located (Table 2).

**Table 2 -:** Not rare pathogenic variants and their strongest gene-disease association according to MalaCards.

Variant	MAF	ABraOM	SilVA	CADD	Gene	Disease (MCID^†^)
						Monogenic
22-38051628-C-A	0.02048	0.045230	0.479	17.10	*PLCD1* ^*§*^	NLD012, LKN007
11-77090939-C-T	0.01339	0.006568	0.333	15.09	*PAK1* ^*§*^	INT331
						Multifactorial
17-7468277-C-T	0.03548	0.022167	0.504	18.72	*SENP3*	PSR032, ATS523, multiple cancers
12-122292609-C-T	0.01160	0.007389	0.590	22.90	*HPD* ^*§*^	HWK001, TYR011
17-80399692-T-G	0.02413	0.022167	0.288	18.50	*HEXDC*	MLD018, AMY004
10-63450379-G-A	0.02376	0.015599	0.445	21.70	CABCOCO1	CNR021
10-56106173-T-C	0.01359	0.021346	0.346	16.76	*PCDH15* ^*§*^	USH041, DFN093
6-46851296-C-T	0.01190	0.020525	0.400	17.54	*ADGRF5*	PLY117, RTN048, SFT011
20-44580788-G-T	0.02123	0.037767	0.344	22.10	*ZNF335* ^*§*^	MCR223
21-33678976-G-T	0.01311	0.003284	0.368	18.28	*MRAP*	GLC043, FML063
19-41086309-G-C	0.03815	0.042693	0.419	16.05	*SHKBP1*	AML050, NNN034, BRK001, DLF001, KRN001, SML004
6-31938120-C-T	0.01684	0.018062	0.362	22.60	*DXO*	CTS005, CRY008
11-86159223-C-T	0.05721	0.061576	0.368	17.66	*ME3*	THY062
12-132416780-C-A	0.01648	0.015599	0.432	16.76	*PUS1* ^*§*^	MYP021
15-64452322-G-A	0.01375	0.010673	0.305	18.60	*PPIB* ^*§*^	OST130, BRT054, OST122, OST121, OST080
16-66764069-G-C	0.01689	0.011494	0.405	16.61	*DYNC1LI2*	BRD019
11-76867135-C-T	0.04401	0.033662	0.312	20.30	*MYO7A* ^§^	USH036, DFN250, DFN251, USH001, RRG078, RTN008, SNS001, USH035, FND002, NNS072, CNR004, NNS044, ERM002, RRT027, RRT028
9-86354657-A-C	0.01474	0.002463	0.360	15.01	*GKAP1*	multiple cancers
16-67917958-G-A	0.03065	0.044335	0.316	22.00	*EDC4*	ULN001, THR013
4-113468564-C-T	0.05138	0.030378	0.309	22.20	*ZGRF1*	ISL163
13-30107118-A-C	0.04922	0.022167	0.359	15.66	*SLC7A1*	HYP595
19-39329205-C-T	0.02172	0.025452	0.352	20.90	*HNRNPL*	AZS001, MTH009
X-153176369-T-C	0.01101	-	0.339	18.67	*ARHGAP4*	NPH007, DBT005, XLN251
3-58376875-T-G	0.01735	-	0.316	15.25	*PXK* ^§^	SYS001
13-51941943-T-C	0.01705	0.009852	0.317	18.81	*INTS6*	multiple cancers
13-46942949-A-C	0.1767	0.041051	0.656	16.39	*RUBCNL*	multiple cancers
15-81294774-G-C	0.05068	0.040230	0.339	15.71	*TLNRD1*	BLD134
18-43604634-C-T	0.01421	0.006568	0.339	15.60	*PSTPIP2*	CHR288, SPH001
1-54704829-T-C	0.01544	0.032841	0.397	16.85	*SSBP3*	LSS002, CCK001
5-90459600-T-G	0.02762	0.004105	0.388	15.77	*ADGRV1* ^§^	USH020, FBR069, USH001, USH035, FND002, RRG078, EPL140, USH037, GNR002
7-89937168-G-A	0.02964	0.008210	0.817	23.50	*CFAP69* ^§^	SPR127, NNS033
16-4487486-G-A	0.01564	0.004926	0.705	19.68	*DNAJA3*	CDS002, MYS074, ALT004
						Both
7-117199709-G-A	0.01672	0.027094	0.543	23.10	*CFTR* ^§^	CYS001, PNC108, HRD234, BRN076, VSD002, MLN007, PRS050, SPR093, AQG005, IDP074, NCH001, MLN084
3-143371201-C-T	0.03362	0.050082	0.417	22.00	*SLC9A9* ^§^	ATS377, CLR023
19-11325229-C-T	0.01410	0.029557	0.845	23.00	*DOCK6* ^§^	ADM007, FML021
17-33477242-G-A	0.06422	0.046798	0.372	21.90	*UNC45B* ^§^	CTR144, MYP004, MYF012, ERL036, ERL043
14-23856861-C-T	0.01165	0.018883	0.319	22.00	*MYH6* ^§^	CRD089, CRD096, ATR022, SCK022, CRD086, DLT002, HRT038, PTN001, CRD233, FML304, FML272
11-6650684-T-A	0.009917	0.009852	0.307	22.70	*DCHS1* ^§^	VNM003, MTR077, MTR080
15-91543131-T-A	0.01862	0.047619	0.681	22.90	*VPS33B* ^§^	ART062, KRT080, CHL193

† MalaCards ID. For the complete description of disease names, refer to Table S3.

§ Gene is likely to be associated with causing the disease(s), since their gene-disease associations are supported by manually curated and trustworthy sources.

### *FOXD4L5* carries the best candidate for positive selection in the NWE population 

We then decided to evaluate if non deleterious sSNVs were subject to evolutionary constraints, given they comprise the majority of our data. Positive selection acting upon variants classified as benign by both SilVA and CADD (1,243,191 variants) was investigated using the population branch statistics (PBS) to pairwise compare the F_st_ values of variants from two groups of closely related populations against a distant one. In one group (EAS-SAS) we compared East and South Asians, and in the other group (NWE-SEU), we compared North-Western and Southern Europeans, both using African/African American as the outgroup population. PBS is an F_st_-based method with good power to detect recent selection by measuring alleles with extreme frequency in a specific population when compared to two other populations (Yi *et al*., 2010). Variants showing extreme PBS values - the outliers - represent strong candidates for positive selection.

**Figure 3 -:**
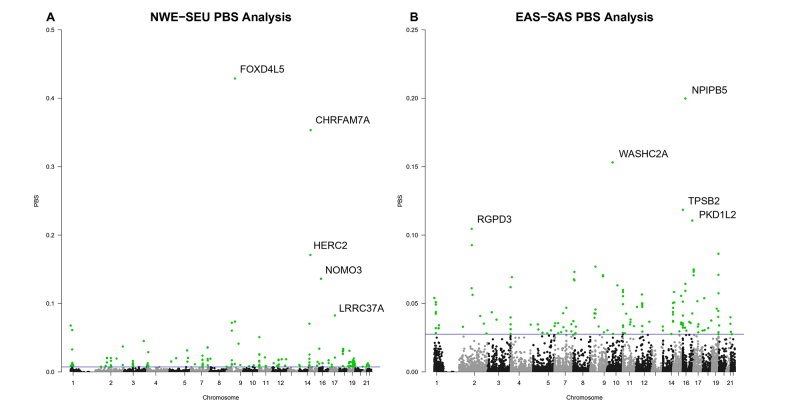
Manhattan plot showing PBS statistics. **A.** PBS outlier analysis for the NWE-SEU group. Highlighted in green, the outliers featuring the top 0.1% best candidates for positive selection. **B.** PBS outlier analysis for the EAS-SAS group. Highlighted in green, the outliers featuring the top 0.1% best candidates for positive selection.

The top 0.1% higher F_st_ values between the NWE-SEU group encompassed 168 sSNVs (Figure 3A ), while 145 sSNVs were found in the top 0.1% F_st_ of the EAS-SAS group (Figure 3B). The two overall best outlier candidates are found to be positively selected on the North-Western European population: a C>T change (PBS = 0.42) on the gene *FOXD4L5* and an A>G change (PBS = 0.35) on the gene *CHRFAM7A* (Table 3). The other three candidates to complete the top 5 for the North-Western European population are the T>C variant (PBS = 0.17) situated on the gene *HERC2*; a T>C variant found in the Nodal Modulator 3 (*NOMO3*) gene (PBS = 0.13); and the G>A sSNV (PBS = 0.08) located on the gene Leucine Rich Repeat Containing 37A (*LRRC37A*).

**Table 3:** - PBS outliers and their genes.

Group	Variant	PBS	Gene	Description
SEU-NWE	9-70177312-C-T	0.428844876	*FOXD4L5*	Forkhead Box D4 Like 5
	15-30654889-A-G	0.353300602	*CHRFAM7A*	Fusion of CHRNA7 and FAM7A
	15-28467246-T-C	0.170901254	*HERC2*	*HECT* And *RLD* Domain Cont. E3 Ubiq. Prot. Ligase 2
	16-16367702-T-C	0.136165561	*NOMO3*	NODAL Modulator 3
	17-44408795-G-A	0.082536940	*LRRC37A*	Leucine Rich Repeat Containing 37A
SAS-EAS	16-22547298-G-T	0.19979496	*NPIPB5*	Nuclear Pore Complex Interacting Protein B5
	10-47911591-T-G	0.15311104	*WASHC2A*	*WASH* Complex Subunit 2A
	16-1279253-T-C	0.11847263	*TPSB2*	Tryptase Beta 2
	16-81242151-T-C	0.11058199	*PKD1L2*	Polycystic Kidney Disease 1-Like 2
	2-107040985-T-C	0.10454195	*RGPD3*	*RANBP2* Like And GRIP Domain Containing 3

### *A G>T change on NPIPB5* is a great candidate for positive selection in the EAS population

The third highest scoring sSNV overall was found to be positively selected on the East Asian population. The G>T change (PBS = 0.19) is located on the gene *NPIPB5* on chromosome 16. The other four variants to complete the five best candidates to be positively selected in the East Asian population, in relevance order, are: the T>G variant (PBS = 0.15), found in the gene *WASHC2A*; the T>C variant (PBS = 0.11) located on the gene Tryptase β 2 (TPSB2); a T>C change (PBS = 0.11) on the gene Polycystic Kidney Disease 1-Like 2 (*PKD1L2)*; the T>C variant (PBS = 0.10) situated on the gene *RGPD3*.

We ran the same groups of populations on Bayescan (v2.1) (Foll and Gaggiotti, 2008), which identifies variants under natural selection using a Bayesian model for estimation of locus-population-specific F_st_ coefficients. Bayescan was not able to identify any outliers (Figure S5), maybe because of high F_st_ values and the small number of populations. This method is known for being conservative, which allows for a low false positive rate (Narum and Hess, 2011), although robustness decreases when the number of screened populations is low (Tigano *et al*., 2017).

## Discussion

Synonymous variants are often considered to be neutral. However, they may impact phenotype through different mechanisms. For example, synonymous variants may have an impact on splicing, through splice consensus disruption or leading to alternative splicing. But, it still might be pathogenic, even if no effect on splice consensus sites or alternate splicing are predicted. However, such variants would probably be classified as Likely Benign with computational support. Moreover, if splicing impact is suspected or evidence hints at pathogenicity, the American College of Medical Genetics’ (ACMG) recommends it should be classified as uncertain until functional evaluation or further evidence is available (Richards *et al*., 2015).

This proposed framework holds potential in the reclassification of synonymous Variants of Uncertain Significance (VUS). Here, we leveraged comprehensive genomic data from multiple populations, allowing for a more extensive assessment of the potential impact of sSNVs. By employing this approach, our study contributes with valuable insights into the functional consequences of these variants, thereby offering a promising avenue for the reclassification of VUS. Through the integration of comprehensive population data and advanced genomic analysis, this method holds promise in enhancing our understanding of VUS and their potential association with diseases. Nonetheless, the utilization of this extensive analysis within a diagnostic context remains ambiguous; conceivably, its application could be tailored for focused reclassification endeavors in specific disease-associated genes.

The results show that, among CADD’s top 5 scoring variants, sSNV 9-139685876-G-A stands out as the sole variant predicted as likely pathogenic by SilVA. This variant has no clinical significance reported in literature yet, and is located on the transmembrane protein 141 (*TMEM141*), on chromosome 9. Although its function is not clearly known, it is speculated that *TMEM141* might be involved in signal transduction across the membrane due to its loosely packed helices (Klammt *et al*., 2012). The other four variants that constitute CADD’s top 5 were deemed potentially pathogenic by SilVA: 19-7747163-G-T, 20-35807771-G-A, 3-73047268-G-A, 9-35809402-G-C. Despite none of them having clinical significance reported, all are located on disease associated genes (Table 1).

Another noteworthy result is that four of SilVA’s top 5 variants are encompassed within the 1% of variants predicted by CADD to be the most deleterious. All of them are located on genes which have been associated with disorders such as cancer, neurological and neurodegenerative diseases. Despite having no strong association with any disease according to MalaCards (Rappaport *et al*., 2013), The Negative Elongation Factor Complex Member E (*NELF-E)* was found to be involved in the regulation of HIV’s post infection transcription (Rao *et al*., 2006). It is also important to observe that none of SilVA’s top 5 scoring sSNVs have clinical significance reported in the literature yet.

The Ensemble Feature Selection analysis revealed that the most relevant feature for the effect prediction is “consequence”, denoting the variant’s impact on genomic features as provided by VEP. Notably, 25% of deleterious variants manifest beyond splicing sites, thus indicating that other mechanisms leading to deleteriousness are at play, such as impaired mRNA stability and codon usage bias. The mechanisms by which a synonymous variant can impair mRNA stability, thus, the efficiency of translation, are not readily discernible. Although there’s a possibility that it could impact translation initiation, it’s also feasible that sSNVs might induce a broader disruption in mRNP structure (Duan *et al*., 2003). When it comes to codon usage bias, it is directly associated with the optimization of fundamental cellular processes, such as speed and accuracy of translation (Hunt *et al*., 2014).

The remaining attributes within the top eight encompass conservation scores provided by various software tools. Conservation is one of the most important aspects for assessing the effect of any variant. Evolutionary processes have discarded most deleterious mutations, albeit variants of all levels of conservation are required for species adaptability (Miller *et al*., 2019). “Conservation” is also related to the non-random frequency of synonymous codon selection that varies from organism to organism, and even from gene to gene. The change of the preferred codon for an unusual one can influence the efficiency of gene expression processes and folding of the protein (Spencer *et al*., 2012). The significance of the proportion of heterochromatin state in 127 cell types, ranked ninth in our analysis, can be attributed to the nature of heterochromatin, primarily comprising inactive regions of the genome where gene expression is limited in most instances.

When categorizing the variants based on their allele frequency, it is important to consider the underlying assumption that detrimental variants generally exhibit low AF due to evolutionary processes (Buhr *et al*., 2016). Therefore, variant effect predictors lean to filter deleterious variants of higher frequency and neutral variants of lower frequency (Zeng and Bromberg, 2019). This contributes to the observation of a substantial percentage of variants from both classes, benign and deleterious, possessing an AF ≤ 0.1%, thereby being classified as super rare in this study.

The results of enrichment analyses for the 9635 genes where the super rare variants are situated indicate that metabolic pathways are significantly enriched in a substantial number of genes. However, it is noteworthy that these enriched pathways represent less than 35% of the total gene set associated with them. Although it is possible that many sSNVs may play a role in the diversity of metabolic pathways, it is also reasonable to assume that this is a very unspecific pathway, overrepresented in too many genes. The sSNV 6-152631823-C-T, one of the top-scoring SilVA variants, is located on the gene SYNE1, which has been associated with spinocerebellar ataxia. This pathway was found to be enriched in 66 genes. This analysis might reveal new targets for disease linkage due to its strength of association (FDR = 2.76x10−9).

Regarding the not rare deleterious variants, the findings reveal that all 39 sSNVs are situated within genes linked to one or more diseases, except for *GAKP1*. Interestingly, a missense variant in this gene was shared by three esophageal squamous cell carcinoma (ESCC) patients in a study that aimed to identify candidate susceptibility variants for ESCC (Donner *et al*., 2017). This gene encodes a protein that is highly similar to the mouse *GKAP1* protein. In mice, *GKAP1* is known to be mainly expressed in the testis, and its deletion increases sperm production (Wang *et al*., 2019).

The results for the positive selection analyses revealed that our best candidate for positive selection was found on the gene *FOXD4L5*, located on chromosome 9. Gene ontology annotations for the *FOX* family gene member predict it to enable transcription factor-DNA binding. *FOXD4L5* was found to be one of the top 12 highly mutated genes in a familial lung cancer study (Kanwal *et al*., 2018), although further investigation is necessary to assess whether the mutations contribute to the development of cancer. Importantly, the sSNV that we found on *FOXD4L5* was not one of those mutations and has never been associated with diseases to date.

The second PBS highest scoring variant overall is an A>G change and it’s located on the gene *CHRFAM7A*, on chromosome 15. This gene is the result of the duplication of exons 5 to 10 of *CHRNA7* in fusion with *FAM7A*, a cluster of seven exons (A to F) located both upstream and downstream of *CHRNA7* on chromosome 15 (Di Lascio *et al*., 2022). Although no clinical significance has yet been reported for this variant, *CHRFAM7A* has been widely associated with neurological disorders such as schizophrenia and bipolar disorder (Kunii *et al*., 2015). Further studies in leukocytes and macrophages revealed that *CHRFAM7A* plays an important role in the activation of the cholinergic anti-inflammatory pathway (Maroli *et al*., 2019).

The T>C variant is the third best candidate to be positively selected in the North-Western European population. No clinical significance has been reported to this variant. It is found on the gene *HERC2*, on chromosome 15, which belongs to the *HERC* gene family. This family is known to produce large proteins with multiple structural domains. *HERC2* is related to ligase activity and ubiquitin protein ligase binding according to gene ontology annotations and was recently found to play an important role on the regulation of nucleolar localization of the helicases (Zhu *et al*., 2021). *HERC2* is frequently downregulated in numerous types of cancers due to its critical role on chromosomal stability (Wu *et al*., 2018), and variants in this gene have been associated with developmental delay (Puffenberger *et al*., 2012).

The fourth best candidate to be positively selected on the North-Western European population is a T>C variant found on the gene *NOMO3*, on chromosome 16. It codes for a transmembrane protein that is highly conserved among human tissues (Sun *et al*., 2022) and participates in a complex that takes part in the Nodal signaling pathway during vertebrate development (Haffner *et al*., 2004). A study of a five generation family with Multiple Synostoses Syndrome Type 4 presenting reduction of the *GDF6* gene expression reported *NOMO3* as severely downregulated, which might indicate that it plays a role in the GDF6 pathway to skeletal joint development and ossification (Clarke *et al*., 2021). An immune profiling of Medullary Thyroid Cancer (MTC) reported *NOMO3* to be highly expressed in MTC, configuring as potential tumor-associated antigen (Pozdeyev *et al*., 2020). Importantly, the sSNV discussed here has no clinical significance reported in the literature.

The G>A sSNV found on the gene *LRRC37A* has no clinical significance reported. The *LRRC37* gene family is located on a complex region on chromosome 17 subject to high linkage disequilibrium (Bekpen *et al*., 2012). Several studies reported that genes located in this region, such as *LRRC37*, are associated with Parkinson Disease risk (Bowles *et al*., 2022). Furthermore, Bowles *et al*. (2022) demonstrated that *LRRC37A* produces a membrane-associated protein that contributes in chemotaxis, astroglial inflammation and cellular migration.

The aforementioned sSNVs exhibited positive selection in the north-western European population. Notably, the overall third highest scoring sSNV demonstrated positive selection in the East Asian population. This particular variant involves a G>T change and is situated on the gene NPIPB5, which is located on chromosome 16. No clinical significance has been reported to this variant, but a recent study described the gene *NPIPB5* as a putative novel prognostic biomarker for clear cell renal cell carcinoma (Wang *et al*., 2022).

On the gene *WASHC2A*, we find the T>G variant, a good candidate for positive selection on the East Asian population, with no association with diseases to date. The *WASHC2A* gene, located on chromosome 10, is part of the gene family *FAM21* which is the largest component of the multiprotein complex called The Wiskott-Aldrich syndrome protein and SCAR homologue (*WASH*) complex (Lee *et al*., 2016). This complex takes part on the endosomal sorting pathway, where endosomes sort the proteins for lysosome degradation or the recycling pathway (Bonifacino and Rojas, 2006). Depletion of the *WASH* complex results in endosomal sorting defects in subsequent pathways (Gomez and Billadeau, 2009). Furthermore, Vincendeau *et al*. (2010) demonstrated that a protein fragment which interacts with the HIV regulatory protein Rev, can control HIV replication and is located in the highly conserved proteins encoded by *FAM21* genes.

Another noteworthy variant is the T>C change, located on the gene *TPSB2*, on chromosome 16. β tryptases are tetrameric serine proteases secreted by mast cells upon activation (Schwartz and Irani, 2000). Elevated serum level of mature Tryptase β serves as diagnostics for mastocytosis (Valent *et al*., 2001).

The fourth sSNV most likely to be positively selected in the East Asian population is a T>C change on the gene *PKD1L2*, located on chromosome 16. This gene encodes a protein member of the polycystin family, which is composed of membrane proteins that function as ion-channel regulators and share significant homology to each other (Yuasa *et al*., 2004). More specifically, the product of *PKD1L2* has several alternative splicing forms and binds to specific G-protein subunits, which are responsible for transducing extracellular signals into the cell (Yuasa *et al*., 2004). Besides being associated with polycystic kidney disease, a study in the Korean population revealed that a copy number variation in *PKD1L2* is related with colorectal cancer predisposition (Park *et al*., 2017). Additionally, *PKD1L2* features a four-mRNA model recently constructed for prediction of breast cancer prognosis (Qi *et al*., 2019).

Finally, the T>C variant is our last candidate for positive selection on the East Asian population. It is situated on the gene *RGPD3*, a member of the *RGP* gene family, located in the cluster of Ran-binding protein-related genes, on chromosome 2. This gene family is composed of eight partial copies of the *RanBP2* gene, originating from intrachromosomal segmental duplication, with the posterior acquisition of the GRIP domain. It is likely that *RGP* genes play a role in intracellular trafficking (Ciccarelli *et al*., 2005). Moreover, a study on what influences craniofacial morphology on Northern Han Chinese suggested that the *RGPD3* gene is associated with the morphology of the nose and ears (Wu *et al*., 2019).

It is significant to point out that all positively selected sSNV here described have no clinical significance reported in the literature. Most are located on functionally important, pleiotropic genes that may be benefiting from more frequently used codons that confer better pace of synthesis and cotranslational folding of proteins. This fitness benefit would explain why these variants are likely being positively selected in, at least, two populations. However, accurately determining the specific advantages conferred by such positive selection poses a significant challenge due to the prevailing focus of most studies on diseases linked to specific genetic variations, genes or genomic regions. Further investigation taking into account whether causal sSNVs are also subject to evolutionary constraints and/or associated with life-history traits of a population, as well as with diseases, may complement these findings.

The results regarding positive selection must be taken with caution. Most methods used in population genetics were developed considering natural populations, that is, geographically dispersed populations. Here, we have to take into account that gnomAD uses ancestry informative markers (AIM) for the biogeographical classification of populations by training a random forest model using samples with known ancestry (Karczewski *et al*., 2020). Grouping individuals based on shared characteristics in order to make sense of the biological variation is a complex task and has many possibilities depending on the population concept being used. The outlier analysis can also be affected by the different number of samples in each population (see Materials and Methods) and by the lack of genotype level information on the gnomAD files. We were not able to use data from the 1000 Genomes Project to access genotype information in this analysis as roughly only 20% of the sSNVs present in the gnomAD data were present at the 1000 Genomes database, although it comprises most of gnomAD samples for which exome sequencing is available.

In this study, two sophisticated predictors have been employed, both of which incorporate a range of features and mechanisms to comprehensively assess the potential impact of sSNVs on the human exome. The resulting framework provides a highly detailed and accurate prediction of the effect of these variants on gene function. Vihinen (2022) suggested ”unsense” as a new nomenclature for synonymous variants having an effect on the protein. This may fundamentally change the way in which predictors annotate these variants in the future. Therefore, accurately annotating the deleteriousness of synonymous variants is increasingly challenging, and requires a more comprehensive approach that takes into account the diverse mechanisms by which these variants can affect gene function.

## Conclusions

In this study we have shown that a significant number of sSNVs can have an impact on protein function and propose a framework for sSNVs variant prioritization. This effect seems to be related mainly to alteration of splice sites and loss of preferred codons, suggesting an effect on protein folding. The fact that a substantial portion of the predicted deleterious sSNVs have ultra rare allelic frequency in the population and are present in disease-related genes strengthens our predictions. Finally, outlier analyses revealed that at least ten benign sSNVs are likely to be under positive selection in two populations. Taken together these results give voice to the so-called silent mutations and caution that these variants must be included in variant prioritization analysis. As with any prediction study, further experimental confirmation should be advised.

## Supplementary material

The following online material is available for this article:

Figure S1 - Dataset schematic view. Proportion of synonymous variants compared to the initial downloaded dataset, and proportion of synonymous variants in both classes (benign and deleterious).

Figure S2 - Classification of the variants according to the allele frequency.

Figure S3 - Manhattan plots showing the results for the Bayescan analysis.

Figure S4 - Gene Ontology (GO) enrichment analysis of the genes in which the super rare deleterious variants are located.

Figure S5 - Gene Ontology (GO) enrichment analysis of the genes in which the rare deleterious variants are located.

Table S1 - Ranking of CADD relevant features from the Ensemble Feature Selection analysis and the description of the features (Rentzsch , 2019).

Table S2 - Description of CADD features removed from the Ensemble Feature Selection analysis.

Table S3 - Name description of diseases from Table 2.

File S1 - Tab-separated file (.txt) containing all sSNVs classified as benign in this study, as well as its relevant features and scores.

File S2 - Tab-separated file (.txt) containing all sSNVs classified as deleterious in this study, as well as its relevant features and scores.

File S3 - Comma-separated file (.csv) showing the results for the KEGG analysis on the super rare deleterious sSNVs.

File S4 - Comma-separated file (.csv) showing the results for the Gene Onothology analysis on the super rare deleterious sSNVs.

File S5 - Comma-separated file (.csv) showing the results for the KEGG analysis on the rare deleterious sSNVs.

File S6 - Comma-separated file (.csv) showing the results for the Gene Onthology analysis on the rare deleterious sSNVs.
