# Supplementary material for: Broken silence: 22,841 predicted deleterious synonymous variants identified in the human exome through computational analysis
Source: Genet Mol Biol. 2024 Jan 22;46(3 Suppl 1):e20230125. doi: 10.1590/1678-4685-GMB-2023-0125 (PMC10804382; doi:10.1590/1678-4685-GMB-2023-0125)
Supplement: Table S1 - [file 1415-4757-GMB-46-03-s1-e20230125-s6.pdf]

Genetics and Molecular Biology
https://doi.org/10.1590/1678-4685-GMB-2023-0125

Supplementary Material to "Broken silence: 22,841 predicted deleterious synonymous variants identified in the human exome through computational analysis"

Table S1 - Ranking of CADD relevant features from the Ensemble Feature Selection analysis and the description of the features (Rentzsch *et al.*, 2019).

Pos	Feature	Description
1	Consequence	VEP consequence, priority selected by potential impact
2	verPhCons	Vertebrate PhastCons conservation score
3	mamPhCons	Mammalian PhastCons conservation score
4	mamPhyloP	Mammalian PhyloP conservation score
5	GerpS	Rejected Substitution score defined by GERP++
6	verPhyloP	Vertebrate PhyloP conservation score
7	priPhyloP	Primate PhyloP conservation score
8	priPhCons	Primate PhastCons conservation score
9	cHmmHet	Proportion of 127 cell types in heterochromatin state
10	oAA	Reference amino acid
11	GerpN	Neutral evolution score defined by GERP++
12	cHmmZnfRpts	Proportion of 127 cell types in cHmmZnfRpts state
13	CpG	Percent CpG in a window of +/- 75bp
14	cHmmTssA	Proportion of 127 cell types in cHmmTssA
15	Exon	Exon number/Total number of exons
16	GC	Percent GC in a window of +/- 75bp
17	bStatistic	Background selection score
18	cHmmReprPC	Proportion of 127 cell types in cHmmReprPC state
19	cHmmQuies	Proportion of 127 cell types in cHmmQuies state
20	cHmmTssAFlnk	Proportion of 127 cell types in cHmmTssAFlnk state

Pos	Feature	Description
21	Rare10000bp	Number of rare (MAF <0.05) gnomAD SNV in 10000 bp window nearby
22	cHmmReprPCWk	Proportion of 127 cell types in cHmmReprPCWk state
23	cHmmTssBiv	Proportion of 127 cell types in cHmmTssBiv state
24	EncH3K4Me3	Maximum ENCODE H3K4 trimethylation level
25	Dist2Mutation	Distance between the closest gnomAD SNV up and downstream (position itself excluded)
26	Sngl100bp	Number of single occurrence gnomAD SNV in 100 bp window nearby (default: 0)
27	Sngl1000bp	Number of single occurrence gnomAD SNV in 1000 bp window nearby
28	EncH3K27Ac	Maximum ENCODE H3K27 acetylation level
29	Segway	Result of genomic segmentation algorithm
30	cHmmBivFlnk	Proportion of 127 cell types in cHmmBivFlnk state
31	cHmmTxFlnk	Proportion of 127 cell types in cHmmTxFlnk state
32	Sngl10000bp	Number of single occurrence gnomAD SNV in 10000 bp window nearby
33	minDistTSE	Distance to closest Transcribed Sequence End (TSE)
34	cDNApos	Base position from transcription start
35	Rare1000bp	Number of rare (MAF <0.05) BRAVO SNV in 1000 bp window nearby
36	CDSpos	Base position from coding start
37	Rare100bp	Number of rare (MAF <0.05) gnomAD SNV in 100 bp window nearby
38	protPos	Amino acid position from coding start
39	EncNucleo	Maximum of ENCODE Nucleosome position track score
40	Pos	Position (1-based)
41	EncH3K4Me1	Maximum ENCODE H3K4 methylation level
42	minDistTSS	Distance to closest Transcribed Sequence Start (TSS)
43	Freq 10000 Bp	Number of frequent (MAF >0.05) BRAVO SNV in 10000 bp window nearby
44	cHmmEnh	Proportion of 127 cell types in cHmmEnh state
45	cHmmTxWk	Proportion of 127 cell types in cHmmTxWk state

Pos	Feature	Description
46	cHmmEnhG	Proportion of 127 cell types in cHmmEnhG state
47	Freq1000bp	Number of frequent (MAF >0.05) gnomAD SNV in 1000 bp window nearby
48	cHmmTx	Proportion of 127 cell types in cHmmTx state
49	cHmmEnhBiv	Proportion of 127 cell types in cHmmEnhBiv state
50	Freq100bp	Number of frequent (MAF >0.05) gnomAD SNV in 100 bp window nearby
51	relProtPos	Relative position in protein codon
52	relcDNApos	Relative position in transcript
53	relCDSpos	Relative position in coding sequence

Reference

Rentzsch P, Witten D, Cooper GM, Shendure J and Kircher M (2019) CADD: Predicting the deleteriousness of variants throughout the human genome. Nucleic Acids Res 47:D886-D894.
