# Supplementary material for: Broken silence: 22,841 predicted deleterious synonymous variants identified in the human exome through computational analysis
Source: Genet Mol Biol. 2024 Jan 22;46(3 Suppl 1):e20230125. doi: 10.1590/1678-4685-GMB-2023-0125 (PMC10804382; doi:10.1590/1678-4685-GMB-2023-0125)
Supplement: Table S2 - [file 1415-4757-GMB-46-03-s1-e20230125-s7.pdf]

Genetics and Molecular Biology
https://doi.org/10.1590/1678-4685-GMB-2023-0125

Supplementary Material to "Broken silence: 22,841 predicted deleterious synonymous variants identified in the human exome through computational analysis"

Table S2 - Description of CADD features removed from the Ensemble Feature Selection analysis (Rentzsch *et al.*, 2019).

Feature	Description
Chr	Chromossome
Ref	Reference allele
Alt	Observed allele
CADDscore	CADD PHRED Score
RawScore	Raw score from the model
GeneID	ENSEMBL GeneID
TranscriptID	ENSEMBL feature ID (Transcript ID or regulatory feature ID)
GeneName	GeneName provided in ENSEMBL annotation
Type	Event type (SNV, DEL, INS)
Length	Number of inserted/deleted bases
AnnoType	CodingTranscript, Intergenic, MotifFeature, NonCodingTranscript, RegulatoryFeature, Transcript
ConsScore	Custom deleterious score assigned to Consequence
ConsDetail	Trimmed VEP consequence prior to simplification
nAA	Amino acid of observed variant (default: unknown)
CCDS	Consensus Coding Sequence ID
motifECount	Total number of overlapping motifs
motifEName	Name of sequence motif the position overlaps
motifEHIPos	Position considered highly informative for an overlapping motif by VEP
motifEScoreChng	VEP score change for the overlapping motif site
Domain	Domain annotation inferred from VEP annotation (ncoils, sigp, lcompl, hmmpanther, ndomain = "other named domain")
Dst2Splice	Distance to splice site in 20bp; positive: exonic, negative: intronic
Dst2SplType	Closest splice site is ACCEPTOR or DONOR

Feature	Description
targetScan	targetscan
mirSVR.Score	MirSVR-Score
mirSVR.E	mirSVR.E
mirSVR.Aln	mirSVR.Aln
GerpRS	Gerp element score
GerpRSpval	Gerp element p-Value
TFBS	Number of different overlapping ChIP transcription factor binding sites
TFBSPeaks	Number of overlapping ChIP transcription factor binding site peaks summed over different cell types/tissue
TFBSPeaksMax	Maximum value of overlapping ChIP transcription factor binding site peaks across cell types/tissue
EncExp	Maximum ENCODE expression value
EncOCC	ENCODE open chromatin code
EncOCCombPVal	ENCODE combined p-Value (PHRED-scale) of Faire, Dnase, polII, CTCF, Myc evidence for open chromatin
EncOCDNasePVal	p-Value (PHRED-scale) of Dnase evidence for open chromatin
EncOCFairePVal	p-Value (PHRED-scale) of Faire evidence for open chromatin
EncOCpolIIPVal	p-Value (PHRED-scale) of polII evidence for open chromatin
EncOCctcfPVal	p-Value (PHRED-scale) of CTCF evidence for open chromatin
EncOCmycPVal	p-Value (PHRED-scale) of Myc evidence for open chromatin
EncOCDNaseSig	Peak signal for Dnase evidence of open chromatin
EncOCFaireSig	Peak signal for Faire evidence of open chromatin
EncOCpolIISig	Peak signal for polII evidence of open chromatin
EncOCctcfSig	Peak signal for CTCF evidence of open chromatin
EncOCmycSig	Peak signal for Myc evidence of open chromatin
dbscSNV.ada score	Adaboost classifier score from dbscSNV
dbscSNV.rf score	Random forest classifier score from dbscSNV

Reference

Rentzsch P, Witten D, Cooper GM, Shendure J and Kircher M (2019) CADD: Predicting the deleteriousness of variants throughout the human genome. Nucleic Acids Res 47:D886-D894.
