# Supplementary material for: Broken silence: 22,841 predicted deleterious synonymous variants identified in the human exome through computational analysis
Source: Genet Mol Biol. 2024 Jan 22;46(3 Suppl 1):e20230125. doi: 10.1590/1678-4685-GMB-2023-0125 (PMC10804382; doi:10.1590/1678-4685-GMB-2023-0125)
Supplement: Table S3 - [file 1415-4757-GMB-46-03-s1-e20230125-s8.pdf]

Genetics and Molecular Biology
https://doi.org/10.1590/1678-4685-GMB-2023-0125

Supplementary Material to “Broken silence: 22,841 predicted deleterious synonymous variants identified in the human exome through computational analysis”

Table S3. Name description of diseases from Table 2.

MCID†	Disease name
NLD012	Nail Disorder, Nonsyndromic Congenital, 3
LKN007	Leukonychia Totalis
INT331	Intellectual Developmental Disorder with Macrocephaly, Seizures, and Speech Delay
PSR032	Psoriasis 11
ATS523	Autosomal Recessive Intellectual Developmental Disorder
HWK001	Hawkinsinuria
TYR011	Tyrosinemia, Type Iii
MLD018	Mild Cognitive Impairment
AMY004	Amyloidosis
CNR021	Cone-Rod Dystrophy 10
USH041	Usher Syndrome, Type if
DFN093	Deafness, Autosomal Recessive 23
PLY117	Polymicrogyria, Bilateral Frontoparietal
RTN048	Retinitis Pigmentosa 19
SFT011	Soft Tissue Chondroma
MCR223	Microcephaly 10, Primary, Autosomal Recessive
GLC043	Glucocorticoid Deficiency 2
FML063	Familial Glucocorticoid Deficiency
AML050	Amelogenesis Imperfecta, Type if
NNN034	Noonan Syndrome 12
BRK001	Brooke-Spiegler Syndrome
DLF001	Dieulafoy Lesion
KRN001	Korean Hemorrhagic Fever
SML004	Small Intestine Neuroendocrine Neoplasm
CTS005	Catastrophic Antiphospholipid Syndrome

MCID†	Disease name
CRY008	Cryopyrin-Associated Periodic Syndrome
THY062	Thyroid Dyshormonogenesis 5
MYP021	Myopathy, Lactic Acidosis, and Sideroblastic Anemia 1
OST130	Osteogenesis Imperfecta, Type Ix
BRT054	Brittle Bone Disorder
OST122	Osteogenesis Imperfecta, Type Iii
OST121	Osteogenesis Imperfecta, Type Iv
OST080	Osteogenesis Imperfecta, Type Ii
BRD019	Bardet-Biedl Syndrome 7
USH036	Usher Syndrome, Type I
DFN250	Deafness, Autosomal Recessive 2
DFN251	Deafness, Autosomal Dominant 11
USH001	Usher Syndrome
RRG078	Rare Genetic Deafness
RTN008	Retinitis Pigmentosa
SNS001	Sensorineural Hearing Loss
USH035	Usher Syndrome Type 2
FND002	Fundus Dystrophy
NNS072	Nonsyndromic Hearing Loss
CNR004	Cone-Rod Dystrophy 2
NNS044	Non-Syndromic Genetic Deafness
ERM002	Ear Malformation
RRT027	Rare Autosomal Dominant Non-Syndromic Sensorineural Deafness Type Dfna
RRT028	Rare Autosomal Recessive Non-Syndromic Sensorineural Deafness Type Dfnb
ULN001	Ulnar Neuropathy
THR013	Thoracic Outlet Syndrome
ISL163	Isolated Childhood Apraxia of Speech
HYP595	Hypertension, Essential
AZS001	Azoospermia
MTH009	Mouth Disease
NPH007	Nephrogenic Diabetes Insipidus
DBT005	Diabetes Insipidus

MCID†	Disease name
XLN251	X-Linked Nephrogenic Diabetes Insipidus
SYS001	Systemic Lupus Erythematosus
BLD134	Bladder Cancer
CHR288	Chronic Recurrent Multifocal Osteomyelitis
SPH001	Sapho Syndrome
LSS002	Lissencephaly
CCK001	Cockayne Syndrome
USH020	Usher Syndrome, Type Iic
FBR069	Febrile Seizures, Familial, 4
USH001	Usher Syndrome
USH035	Usher Syndrome Type 2
FND002	Fundus Dystrophy
RRG078	Rare Genetic Deafness
EPL140	Epilepsy, Idiopathic Generalized
USH037	Usher Syndrome, Type Iia
GNR002	Generalized Epilepsy with Febrile Seizures Plus
SPR127	Spermatogenic Failure 24
NNS033	Non-Syndromic Male Infertility Due to Sperm Motility Disorder
CDS002	Codas Syndrome
MYS074	Myasthenic Syndrome, Congenital, 12
ALT004	Alternating Esotropia
CYS001	Cystic Fibrosis
PNC108	Pancreatitis, Hereditary
HRD234	Hereditary Chronic Pancreatitis
BRN076	Bronchiectasis with or Without Elevated Sweat Chloride 1
VSD002	Vas Deferens, Congenital Bilateral Aplasia of
MLN007	Male Infertility
PRS050	Prss1-Related Hereditary Pancreatitis
SPR093	Spermatogenic Failure, Y-Linked, 2
AQG005	Aquagenic Palmoplantar Keratoderma
IDP074	Idiopathic Bronchiectasis
NCH001	Nuchal Bleb, Familial

MCID†	Disease name
MLN084	Male Infertility with Azoospermia or Oligozoospermia Due to Single Gene Mutation
ATS377	Autism 16
CLR023	Colorectal Cancer
ADM007	Adams-Oliver Syndrome 2
FML021	Familial Hypercholesterolemia
CTR144	Cataract 43
MYP004	Myopathy
MYF012	Myofibrillar Myopathy 11
ERL036	Early-Onset Posterior Subcapsular Cataract
ERL043	Early-Onset Nuclear Cataract
CRD089	Cardiomyopathy, Familial Hypertrophic, 14
CRD096	Cardiomyopathy, Dilated, 1ee
ATR022	Atrial Septal Defect 3
SCK022	Sick Sinus Syndrome 3
CRD086	Cardiomyopathy, Familial Hypertrophic, 1
DLT002	Dilated Cardiomyopathy
HRT038	Heart, Malformation of
PTN001	Patent Foramen Ovale
CRD233	Cardiomyopathy, Dilated, 1b
FML304	Familial Isolated Dilated Cardiomyopathy
FML272	Familial Sick Sinus Syndrome
VNM003	Van Maldergem Syndrome 1
MTR077	Mitral Valve Prolapse 2
MTR080	Mitral Valve Prolapse 1
ART062	Arthrogryposis, Renal Dysfunction, and Cholestasis 1
KRT080	Keratoderma-Ichthyosis-Deafness Syndrome, Autosomal Recessive
CHL193	Cholestasis, Progressive Familial Intrahepatic, 12

†MalaCards ID.
