# Supplementary material for: Broken silence: 22,841 predicted deleterious synonymous variants identified in the human exome through computational analysis
Source: Genet Mol Biol. 2024 Jan 22;46(3 Suppl 1):e20230125. doi: 10.1590/1678-4685-GMB-2023-0125 (PMC10804382; doi:10.1590/1678-4685-GMB-2023-0125)
Supplement: File S3 - [file 1415-4757-GMB-46-03-s1-e20230125-s11.zip › gmb-2023-0125_20231221_suppl_11.docx]

Genetics and Molecular Biology
<https://doi.org/10.1590/1678-4685-GMB-2023-0125>

**Supplementary Material to “Broken silence: 22,841 predicted deleterious synonymous variants identified in the human exome through computational analysis”**

**File S3 -** Comma-separated file (.csv) showing the results for the KEGG analysis on the super rare deleterious sSNVs.
